# Papillary Thyroid Carcinoma Arising in a Thyroglossal Duct Cyst: Radiologic and Pathologic Correlation in a Young Adult

**DOI:** 10.7759/cureus.106617

**Published:** 2026-04-07

**Authors:** Bilal Turfe, Alexander M Satei, David Rawls, Rajbir S Pannu, Paul J Arpasi

**Affiliations:** 1 Diagnostic Radiology, Trinity Health Oakland Hospital, Pontiac, USA; 2 Diagnostic Radiology, Wayne State University, Detroit, USA

**Keywords:** computed tomography, ectopic thyroid tissue, midline neck mass, papillary thyroid carcinoma, sistrunk procedure, thyroglossal cyst, thyroglossal duct cyst malignancies, ultrasound, ultrasound-guided biopsy

## Abstract

Thyroglossal duct cysts are the most common congenital cervical anomalies, arising from incomplete involution of the thyroglossal duct during embryologic thyroid descent. Although typically benign, malignant transformation occurs in approximately 1% of cases, most commonly papillary thyroid carcinoma. We report a case of a 27-year-old female presenting with a progressively enlarging midline neck lump. Ultrasound demonstrated a complex cystic lesion superior to the thyroid gland. Contrast-enhanced computed tomography revealed a cystic structure containing a peripherally hyperattenuating, centrally hypoattenuating nodular component with associated punctate calcifications. Ultrasound-guided biopsy confirmed papillary thyroid carcinoma. The patient then underwent total thyroidectomy, with pathology demonstrating a 1.5 cm papillary thyroid carcinoma arising within a thyroglossal duct cyst, without nodal metastases but with positive margins. Histopathology revealed characteristic papillary architecture with fibrovascular cores lined by atypical epithelial cells. Postoperatively, the patient received radioactive iodine (I-131) ablation therapy and remains disease-free at one-year follow-up. This case highlights the importance of recognizing atypical imaging features within thyroglossal duct cysts, which should prompt further evaluation. Accurate diagnosis relies on the correlation of imaging and histopathologic findings. Early detection and appropriate management are essential, as papillary thyroid carcinoma arising in thyroglossal duct cysts carries an excellent prognosis.

## Introduction

Thyroglossal duct cysts (TDCs) represent the most common congenital anomaly of the cervical region. They arise during embryogenesis due to incomplete involution of the thyroglossal duct following the descent of the thyroid gland from the base of the tongue. TDCs are estimated to occur in approximately 7% of the population and demonstrate no clear sex predilection. Although most commonly identified in pediatric patients, up to one-third of cases are diagnosed in adults [1].

Given the presence of ectopic thyroid tissue within the cyst, TDCs have the potential to harbor the same spectrum of malignancies seen in the thyroid gland. Malignant transformation is rare, occurring in approximately 1% of TDCs, with papillary carcinoma accounting for nearly 90% of these cases [2,3]. Less frequently reported malignancies include squamous cell carcinoma and follicular carcinoma [2]. We report a case of papillary thyroid carcinoma arising from a thyroglossal duct cyst in a young female adult.

## Case presentation

A 27-year-old female presented with a progressively worsening sensation of a lump in her throat during swallowing over the preceding several months. The patient denied fever, weight loss, or visible abnormality within the region of concern. Physical examination demonstrated a small palpable mass within the midline of the neck. The patient reported no family history of malignancy. She was a never-smoker and reported occasional alcohol use. Her past medical history was notable for polycystic ovarian syndrome and obesity (body mass index: 50.3 kg/m²).

Given the clinical concern for a thyroid abnormality, baseline thyroid function tests were obtained. Thyroid-stimulating hormone (TSH) was within normal limits at 0.98 μIU/mL (reference range: 0.45-5.33 μIU/mL), and free thyroxine (FT4) was within normal limits at 0.64 ng/dL (reference range: 0.61-1.24 ng/dL). Free triiodothyronine (FT3) and thyroid antibody testing, including anti-thyroid peroxidase and anti-thyroglobulin antibodies, were not performed, as the patient was clinically euthyroid and there was no suspicion for underlying autoimmune thyroid disease based on presentation.

**Figure 1:**
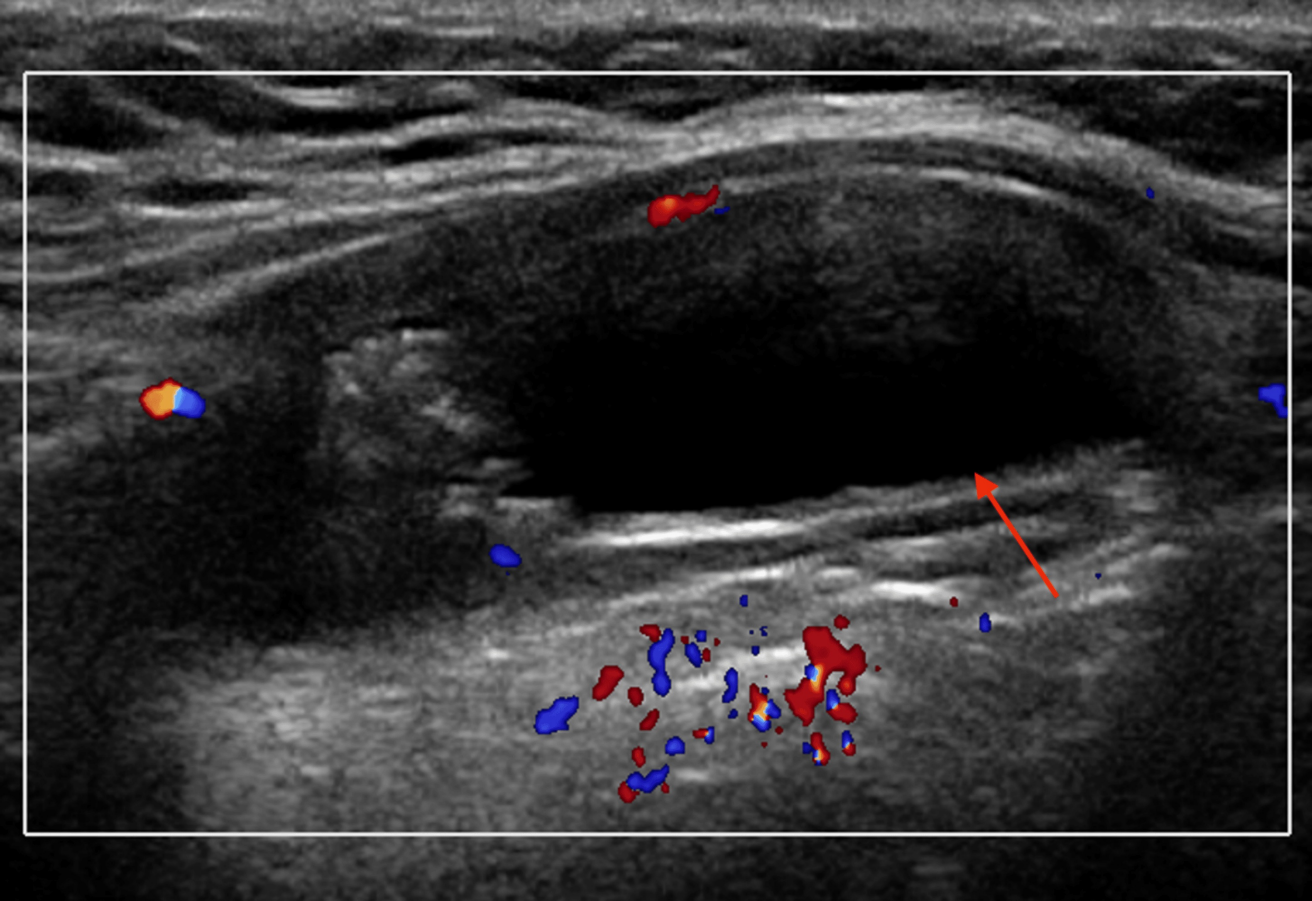
Ultrasound of the neck soft tissues. The ultrasound revealed a complex-appearing cystic structure (red arrow) within the midline of the neck, superior to the thyroid gland and immediately deep to the subcutaneous tissues, measuring 4.1 x 1.4 x 2.1 cm, with no internal vascularity.

**Figure 2:**
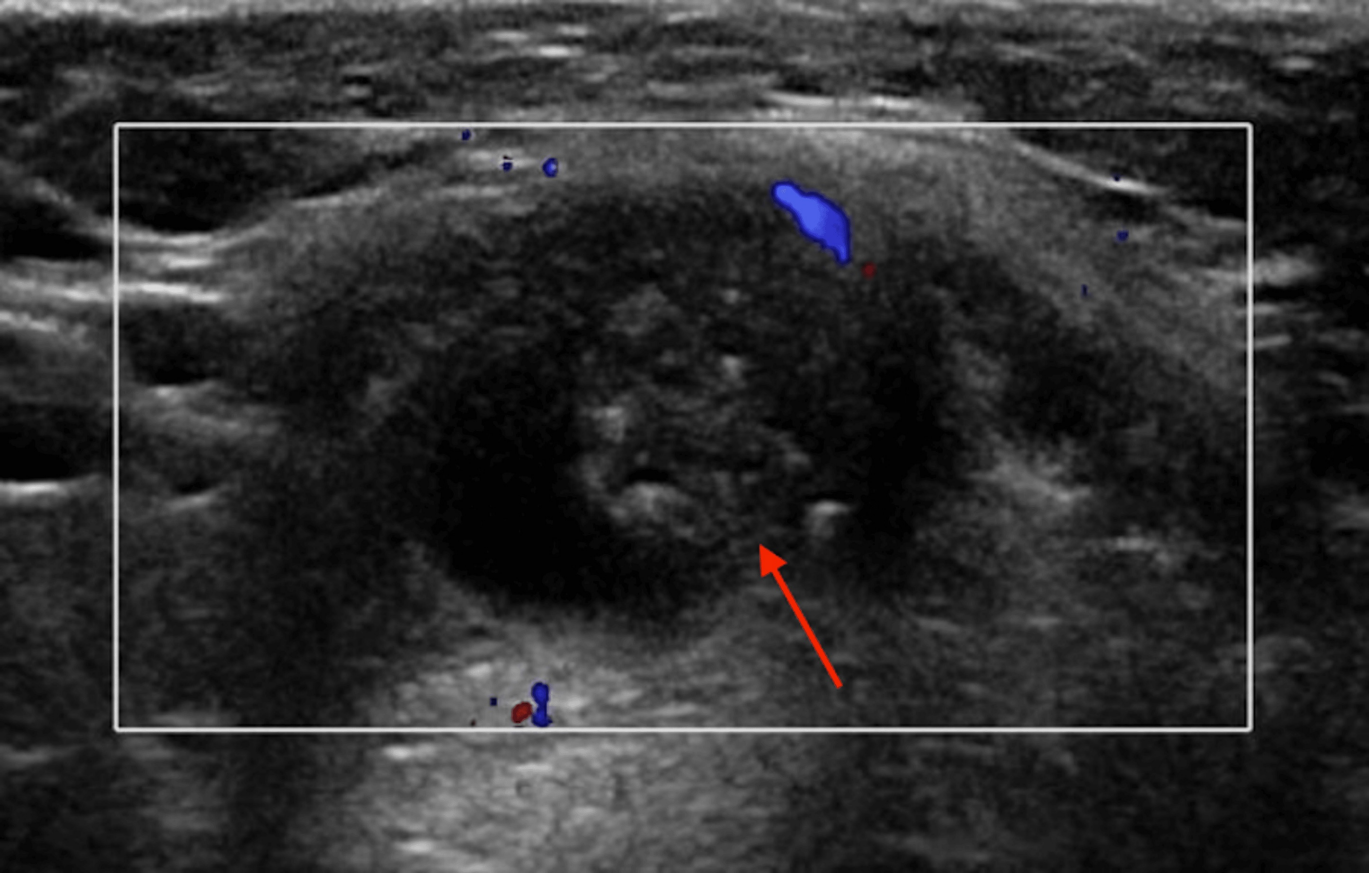
Focused ultrasound of the neck soft tissues. The ultrasound revealed a region of increased echogenicity within the cystic structure, without posterior acoustic shadowing or vascularity (red arrow).

The patient underwent targeted ultrasound of the neck soft tissues, including dedicated evaluation of the thyroid gland. Sonographic assessment of the thyroid demonstrated normal size, echotexture, and vascularity, without focal nodules or suspicious lesions, effectively excluding a primary thyroid abnormality. Sonographic evaluation demonstrated a complex-appearing cystic structure within the midline of the neck, 0.3 cm superior to the thyroid gland and immediately deep to the subcutaneous tissues, measuring 4.1 x 1.4 x 2.1 cm; no internal vascularity was visualized on color Doppler interrogation (Figure 1). A region of increased echogenicity was visualized without posterior acoustic shadowing (Figure 2). There was no sonographic evidence of a direct connection between the lesion and the thyroid gland.

**Figure 3:**
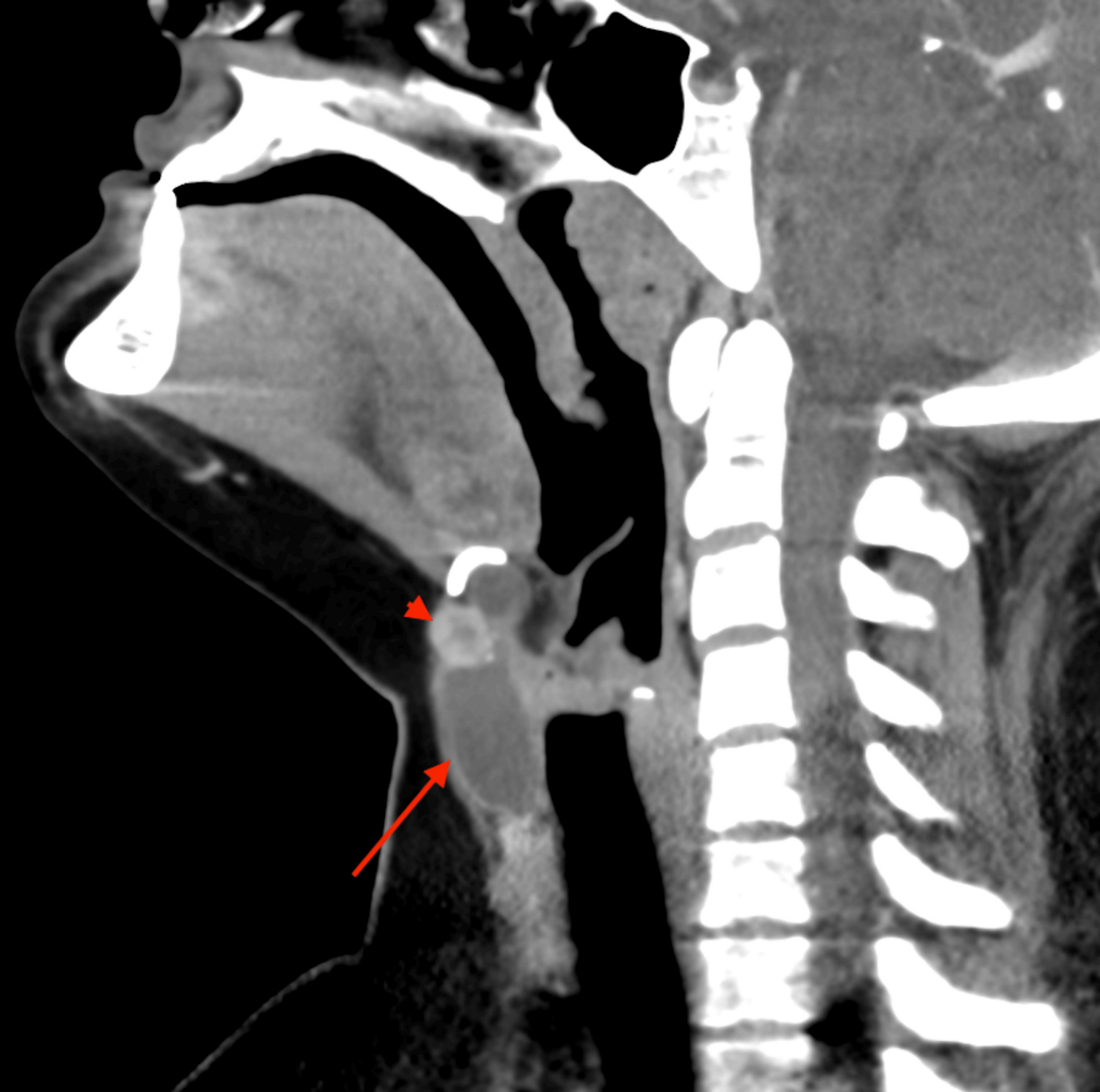
Sagittal CT of the neck with contrast. Sagittal CT revealed a cystic tubular structure (red arrow). There was a peripherally hyperattenuating and centrally hypoattenuating lesion (red arrowhead) in the superior midline aspect of the cyst with multiple adjacent punctate calcifications, measuring 1.0 x 1.2 x 1.3 cm.

**Figure 4:**
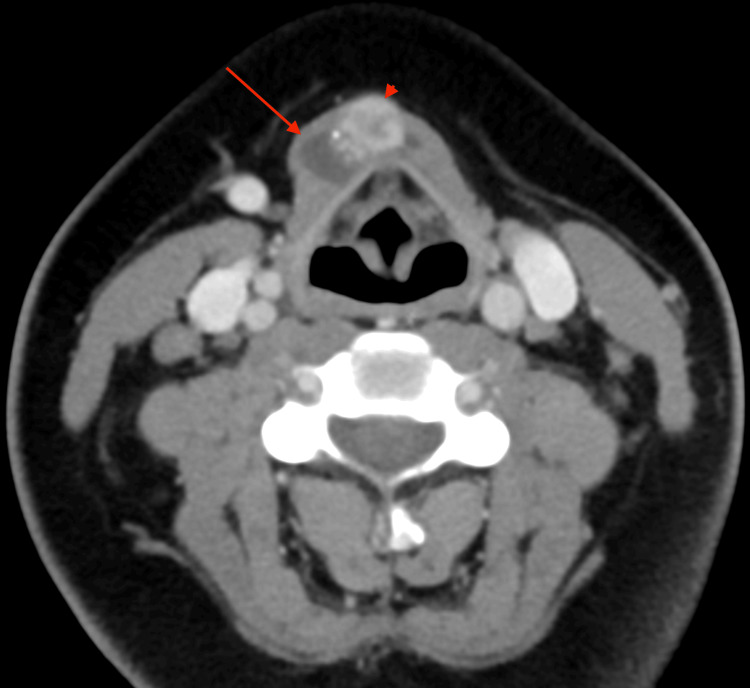
Axial CT of the neck with contrast. Axial CT revealed a cystic tubular structure slightly to the right of the midline (red arrow). There was a peripherally hyperattenuating and centrally hypoattenuating lesion (red arrowhead) in the superior midline aspect of the cyst with multiple adjacent punctate calcifications, measuring 1.0 x 1.2 x 1.3 cm.

Follow-up computed tomography (CT) of the neck with contrast, in sagittal reformat and axial view, demonstrated a cystic and tubular structure slightly to the right of midline, corresponding to the abnormality visualized on ultrasound (Figures 3, 4). There was a peripherally hyperattenuating and centrally hypoattenuating lesion in the superior midline aspect of the cyst with multiple adjacent punctate calcifications measuring 1.0 x 1.2 x 1.3 cm; this finding corresponded to the area of increased echogenicity on ultrasound.

**Figure 5:**
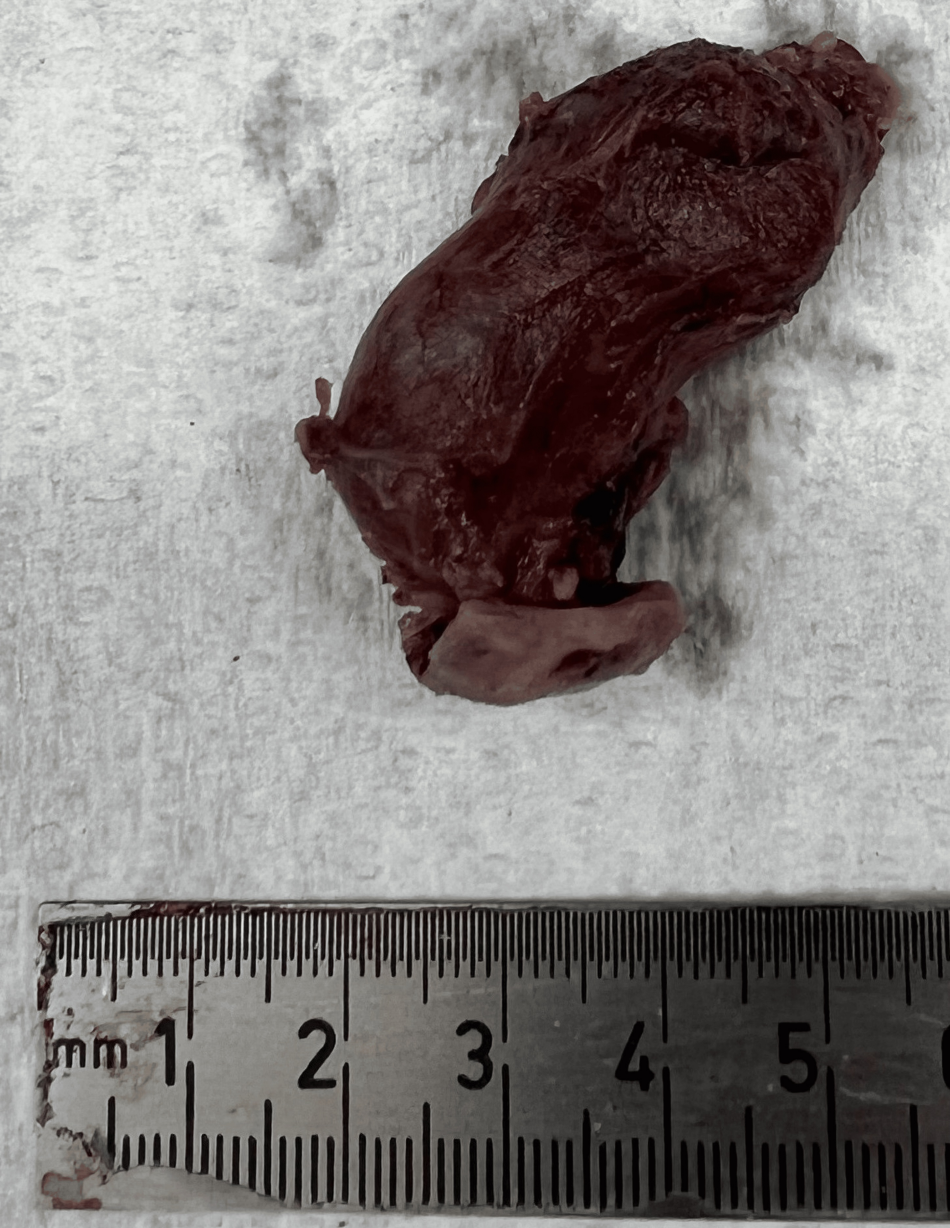
Gross photograph revealed an elongated segment of tan-red thyroid tissue with a smooth external surface, measuring approximately 4-5 cm. This image represents a portion of the resected specimen rather than the entire thyroid gland.

**Figure 6:**
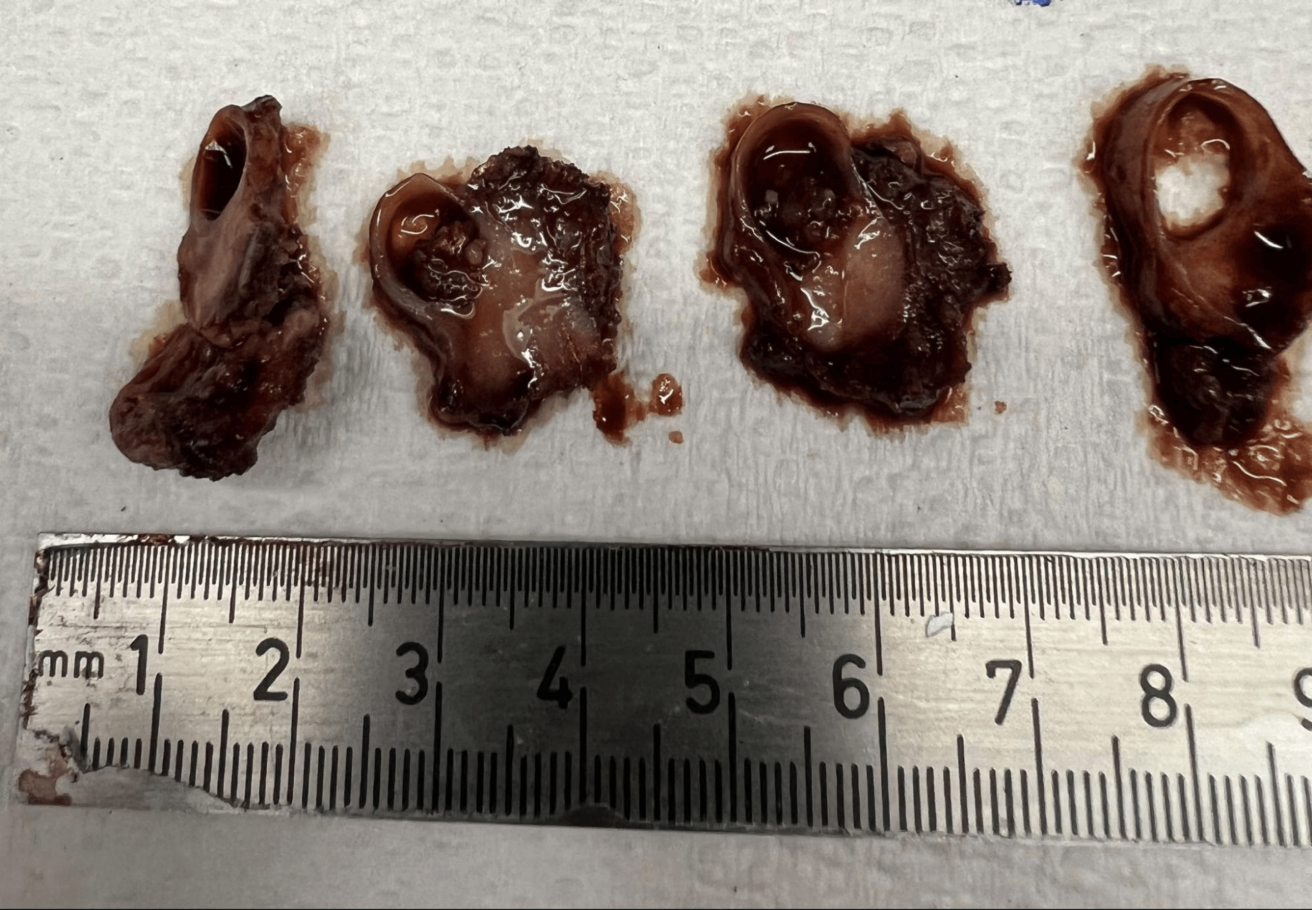
Serial sectioning revealed a cystic structure with a well-defined lumen and a focal intramural solid, tan-white nodular component.

The patient underwent ultrasound-guided biopsy, yielding a diagnosis of papillary thyroid carcinoma. She subsequently underwent total thyroidectomy with excision of the midline cystic lesion. Gross examination demonstrated an elongated segment of resected tissue. Serial sectioning of the lesion revealed a predominantly cystic structure with a well-defined lumen and a thickened cyst wall, from which a firm, tan-white nodular focus projected into the lumen (Figures 5, 6).

**Figure 7:**
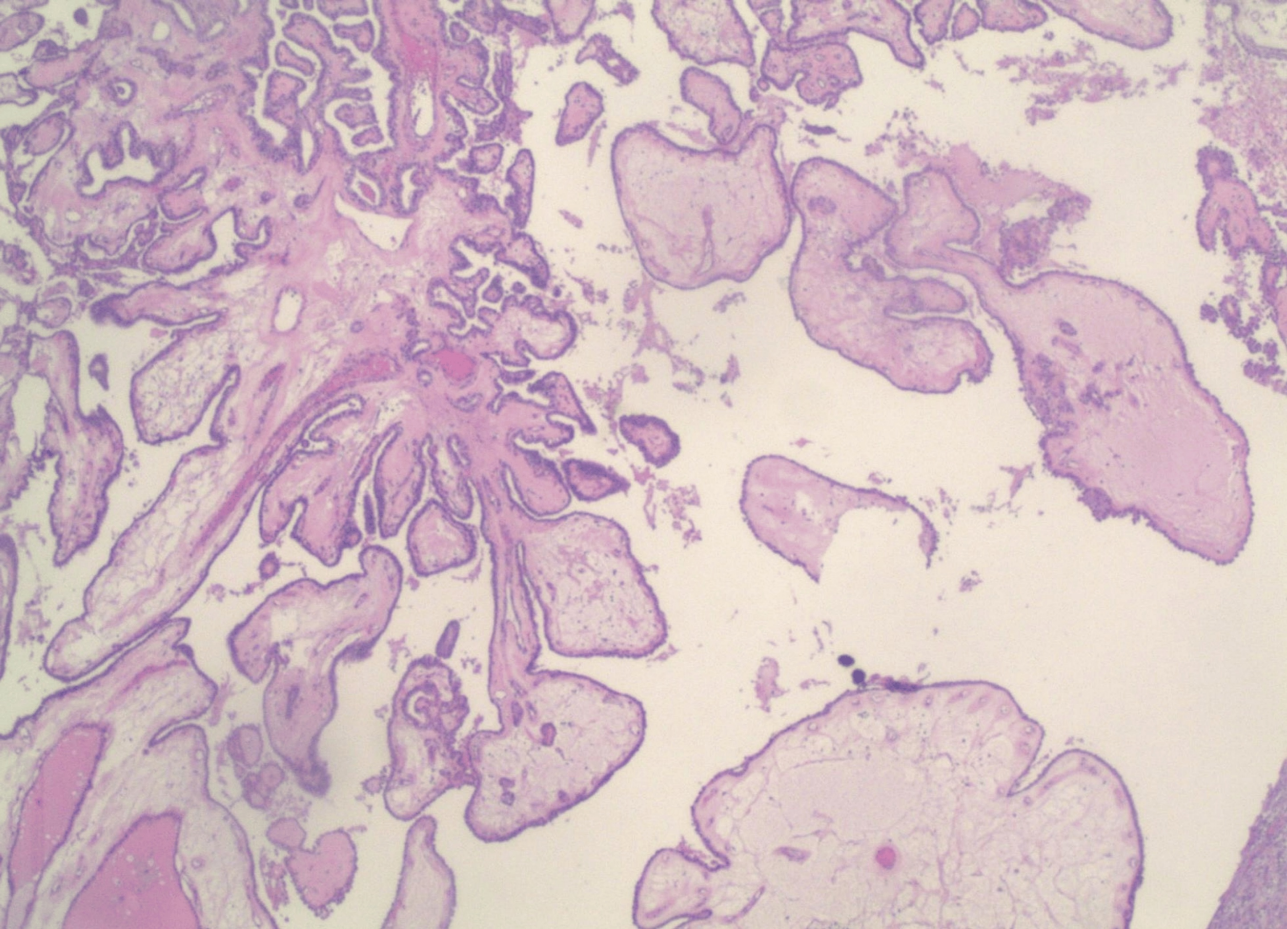
Low-power H&E stain view revealed papillary structures with fibrovascular cores lined by atypical epithelial cells exhibiting characteristic features of papillary thyroid carcinoma.

**Figure 8:**
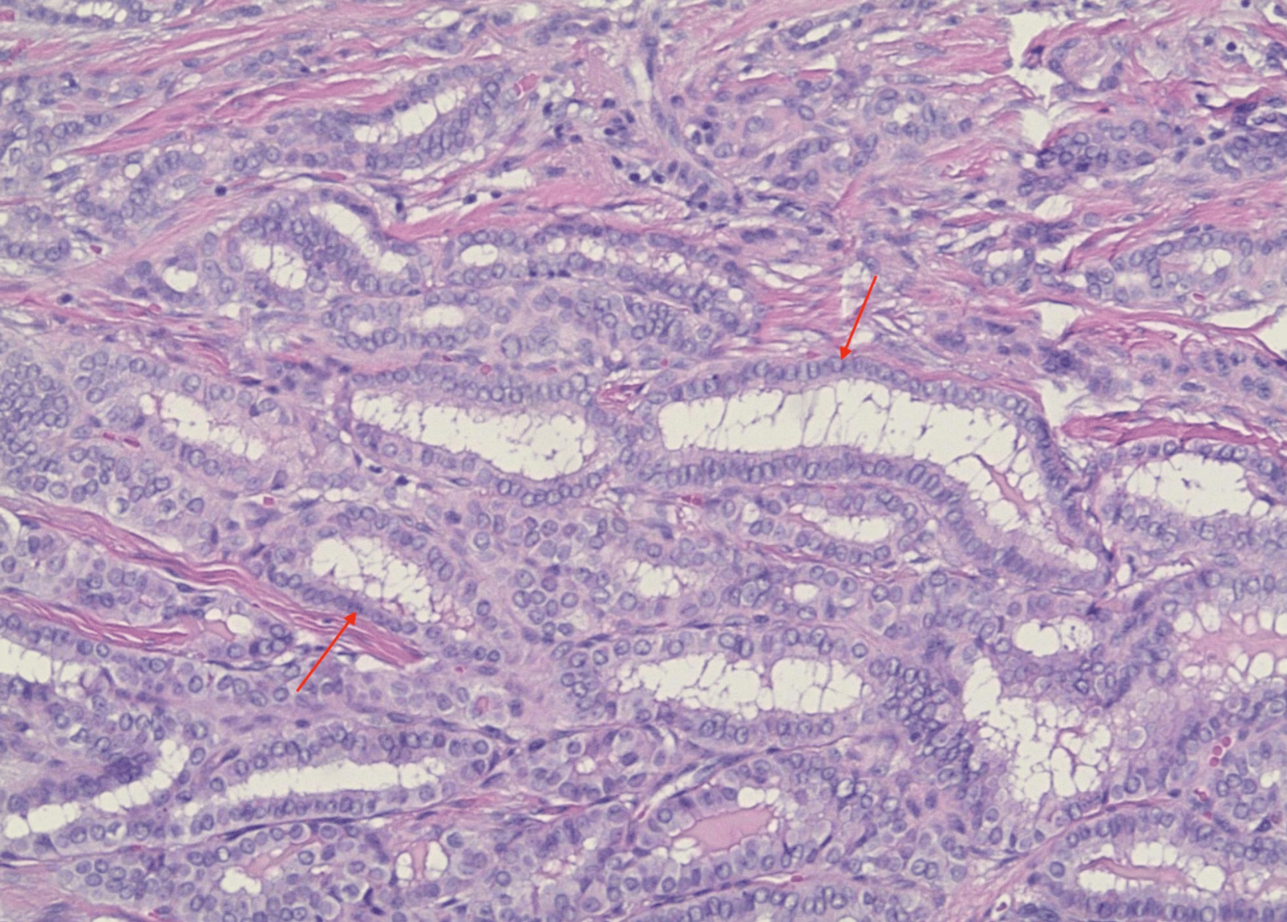
High-power H&E stain view revealed neoplastic thyroid follicles lined by atypical epithelial cells (red arrows) demonstrating nuclear enlargement, overlapping, and chromatin clearing.

The solid component corresponded to the hyperechoic focus identified on preoperative ultrasound imaging. Low- and high-power H&E stains demonstrated papillary architecture composed of branching fibrovascular cores lined by atypical epithelial cells (Figures 7, 8). 

The pathology findings were consistent with papillary thyroid carcinoma measuring 1.5 cm with involvement of the adjacent soft tissue margin. A central neck dissection was performed, with lymphatic tissue obtained from the bilateral central compartment (level VI), including pretracheal and paratracheal regions. All examined lymph nodes were negative for metastatic disease. Importantly, the neoplastic proliferation was centered within and arising from the cyst wall, without involvement of the adjacent thyroid parenchyma. No direct continuity between the lesion and the thyroid gland was identified. These findings support the origin of the carcinoma within a thyroglossal duct cyst rather than a primary thyroid malignancy.

The patient subsequently received oral radioactive iodine (I-131) therapy (31.5 mCi) as part of postsurgical thyroid remnant ablation. This was performed to eliminate residual thyroid tissue and facilitate surveillance for recurrent disease using serum thyroglobulin as a tumor marker. At one-year follow-up, the patient remained asymptomatic, with no clinical evidence of recurrence. The most recent thyroid function tests demonstrated a TSH of 1.65 μIU/mL and FT4 of 0.73 ng/dL, consistent with a euthyroid state on thyroid hormone replacement therapy. Serum thyroglobulin was undetectable (<0.1 ng/mL) and thyroglobulin antibody levels were negative (<1 IU/mL), indicating no biochemical evidence of residual or recurrent disease.

## Discussion

Although thyroglossal duct cyst (TDC) carcinoma is rare, it has been described in several hundred cases in the literature, most commonly as papillary thyroid carcinoma arising within the cyst. No definitive prevalence has been established. Given its rarity, much of the current understanding is derived from case reports and small case series rather than large prospective studies.

Clinically, TDCs typically present as a painless and mobile midline neck mass, most often located at or just inferior to the hyoid bone. In some cases, they may become infected and present as an abscess or intermittently draining sinus [1]. In contrast, papillary thyroid carcinoma generally presents as a painless mass and may be associated with neck swelling and regional lymphadenopathy. Advanced disease can manifest with symptoms such as dysphagia or hoarseness with involvement of the recurrent laryngeal nerve [4].

On ultrasound, TDCs appear as well-circumscribed cystic lesions with posterior acoustic enhancement and displacement of the strap muscles. CT typically demonstrates a thin-walled, well-defined, homogeneously fluid-attenuation lesion in the anterior midline neck. In contrast, papillary carcinoma appears on ultrasound as a solid lesion with irregular margins and punctate echogenic foci consistent with microcalcifications. On CT, papillary carcinoma appears as an irregular and enhancing solid mass which may contain calcifications and cystic components [5,6].

The pathogenesis of papillary carcinoma arising within a TDC remains uncertain. One theory proposes metastatic spread from an occult primary thyroid carcinoma, whereas another supports de novo malignant transformation within ectopic thyroid tissue in the cyst [5]. These differing theories have implications for management. The Sistrunk procedure remains the gold standard for treatment, with reported recurrence rates ranging from 0% to 8% [6]. However, the role of total thyroidectomy in cases where carcinoma appears confined to the TDC remains controversial. While Sistrunk alone may be sufficient in select low-risk patients, additional surgical management may be considered in the presence of high-risk features, including larger tumor size, positive margins, local invasion, or concern for synchronous thyroid malignancy [7]. In this case, the presence of a tumor measuring 1.5 cm with positive surgical margins supported a more comprehensive surgical approach. Taken together, these factors provide a rationale for total thyroidectomy in addition to cyst excision, consistent with an individualized, risk-adapted management strategy described in prior studies. Furthermore, total thyroidectomy facilitated postoperative surveillance through serum thyroglobulin monitoring and enabled the use of radioactive iodine therapy, both of which are important in risk stratification and detection of recurrence.

The radiologic-pathologic correlation in this case is critical in supporting the diagnosis of carcinoma arising within a thyroglossal duct cyst rather than a primary thyroid malignancy. On imaging, the lesion was identified as a midline, predominantly cystic mass located superior to the thyroid gland without evidence of direct continuity with the thyroid parenchyma. The presence of a focal echogenic intralesional component raised suspicion for a solid mural nodule within a cystic lesion. Gross and histopathologic evaluation confirmed a cystic structure with a solid nodular component arising from the cyst wall, consistent with papillary thyroid carcinoma. Importantly, there was no involvement of the thyroid gland on imaging or histopathology. This combination of a midline cystic lesion with an intramural solid component and absence of a primary thyroid lesion supports the diagnosis of thyroglossal duct cyst carcinoma and helps distinguish it from metastatic or primary thyroid-derived disease.

Adjuvant radioactive iodine (I-131) therapy is typically reserved for high-risk patients following thyroidectomy, with the aim of ablating residual thyroid tissue and treating microscopic disease to reduce recurrence risk [8]. Additionally, thyroid-stimulating hormone (TSH) suppression therapy with levothyroxine is used at times to limit stimulation of residual or recurrent tumor cells and has been associated with improved disease control [9]. Current evidence supports a risk-stratification approach, utilizing selective radioactive iodine therapy and individualized TSH suppression strategies to balance oncologic benefit with the risk of overtreatment. I-131 therapy was administered in our case as part of postoperative management following total thyroidectomy. The use of I-131 in thyroglossal duct cyst carcinoma is not universally required but may be considered in the presence of higher-risk features, including tumor size >1 cm, positive surgical margins, or concern for residual microscopic disease. In our patient, the presence of a 1.5 cm tumor with positive margins supported the use of adjuvant radioactive iodine therapy to ablate any residual thyroid tissue and reduce the risk of recurrence.

Papillary thyroid carcinoma is associated with an excellent prognosis, with long-term survival rates exceeding 90% and 10-20 year overall survival between 90% and 97% in large cohort studies [10]. Prognosis is influenced by established risk factors, including patient age, tumor size, extrathyroidal extension, and the presence of distant metastases [10].

The clinical, imaging, and pathologic features in our case are largely consistent with those reported in prior cases of TDC carcinoma. Similar to previously described cases, the patient presented with a midline neck mass, and imaging demonstrated a predominantly cystic lesion with an internal solid component located superior to the thyroid gland [5,6]. Histopathologic evaluation confirmed papillary thyroid carcinoma arising from the cyst wall, which represents the most common histologic subtype reported in TDC malignancies. The absence of a primary thyroid lesion and lack of nodal metastases are also consistent with many reported low-risk cases.

## Conclusions

Papillary thyroid carcinoma arising within a TDC is a rare yet clinically significant entity that may be misdiagnosed as a benign cystic lesion. This case highlights the importance of careful radiologic assessment, particularly the identification of suspicious features such as solid components and microcalcifications, which should prompt further diagnostic workup. Correlation with histopathologic findings remains essential for definitive diagnosis. Management should be individualized, typically involving surgical excision with consideration of adjuvant therapy based on pathologic risk features. Increased awareness among clinicians and diagnosticians is crucial to ensure accurate diagnosis and timely intervention, as this entity carries an excellent prognosis and favorable long-term survival when appropriately treated.
